# Transcriptomic profiling reveals selective attenuation of interferon-associated inflammatory signaling and induction of metallothioneins by a zinc-containing oral rinse

**DOI:** 10.3389/fimmu.2026.1896916

**Published:** 2026-07-27

**Authors:** Xiaoyu Huang, Anna Podesser, Murat İnanç Cengiz, Ronald A. Glabonjat, Reinhard Gruber, Layla Panahipour

**Affiliations:** 1 Department of Oral Biology, University Clinic of Dentistry, Medical University of Vienna, Vienna, Austria; 2 Department of Prosthodontics, The Affiliated Stomatological Hospital of Southwest Medical University, Luzhou, China; 3 Institute of Analytical Chemistry, Faculty of Chemistry, University of Vienna, Vienna, Austria; 4 Department of Periodontology, School of Dental Medicine, University of Bern, Bern, Switzerland

**Keywords:** bulk RNA sequencing, chemokines, inflammation, innate immunity, interferons, metallothionein, mouthwashes, zinc

## Abstract

**Background:**

Oral stromal and epithelial cells contribute to mucosal inflammation through cytokine-induced chemokine production. Essential oil-containing mouth rinses are widely used as adjuncts to oral hygiene, but whether zinc-containing formulations directly modulate host inflammatory signaling remains unclear. This study investigated the effects of a zinc-containing Listerine^®^ formulation on IL-1β/TNF-α-induced inflammatory responses in human oral cells.

**Methods:**

Primary human gingival fibroblasts were stimulated with IL-1β/TNF-α in the presence or absence of Listerine^®^. Transcriptional responses were analyzed by RNA sequencing and validated by quantitative PCR and immunoassays. HSC2 epithelial cells were used to assess selected responses across oral cell types. Phospho-STAT1 immunostaining evaluated canonical STAT1 activation, and ICP–MS determined trace-element composition.

**Results:**

IL-1β/TNF-α induced a broad inflammatory transcriptional program characterized by chemokines, NF-κB-associated mediators, and interferon-associated genes. Listerine^®^ attenuated this response, with strongest effects on *CXCL10, CXCL11, IFIT* family members, *GBP* family members, and *OASL*, whereas classical NF-κB-associated chemokines such as *CXCL1, CXCL2*, and *CXCL8* were less affected. Listerine^®^ also induced a zinc-responsive stress-adaptation signature dominated by metallothioneins. ICP–MS analysis confirmed the presence of zinc in Listerine^®^, and zinc alone partially reproduced the suppression of *CXCL10*. In contrast, individual essential oil constituents failed to recapitulate the anti-inflammatory effects of the complete formulation. Notably, Listerine^®^ did not prevent IFN-γ-induced STAT1 phosphorylation, indicating that suppression of interferon-associated genes was not due to general blockade of canonical STAT1 activation.

**Conclusion:**

These findings identify a previously unrecognized host-modulatory activity of a zinc-containing Listerine^®^ formulation characterized by selective attenuation of interferon-associated inflammatory transcription together with induction of a zinc-responsive metallothionein program.

## Introduction

Inflammation is a central pathological feature of periodontal diseases, which are among the most prevalent chronic inflammatory conditions worldwide (1). Gingivitis, the initial and reversible stage of periodontal disease, is characterized by plaque-induced inflammation of the gingival tissues and can generally be controlled through adequate oral hygiene. In contrast, periodontitis is an irreversible condition associated with progressive destruction of connective tissue and alveolar bone, posing a major therapeutic challenge for patients and clinicians. In addition, inflammatory disorders of the oral mucosa, such as oral mucositis during chemo- and radiotherapy, constitute further clinical situations in which excessive inflammation leads to pain, impaired oral function, and reduced quality of life (2, 3).

In these inflammatory conditions, mouth rinses are frequently used as adjunctive measures to support oral hygiene and symptom control (4, 5). Therapeutic mouth rinses, most notably those containing chlorhexidine, exert their clinical effects primarily through antiseptic activity, thereby indirectly reducing inflammation by lowering the microbial burden (6). However, chlorhexidine is not generally considered a direct modulator of inflammatory responses in host cells (7). Essential oil-containing mouth rinses have also been shown in clinical studies and systematic reviews to reduce plaque accumulation, gingival inflammation, and bleeding on probing when used as adjuncts to mechanical plaque control (5, 8, 9). These clinical benefits are generally attributed to antimicrobial activity and the resulting reduction in dental biofilm. Cosmetic mouth rinses, in contrast, are primarily marketed for breath control and oral freshness and are generally not considered to possess host-modulating biological activity. Nevertheless, some formulations contain ingredients with documented bioactive properties (10), suggesting that these products may exert biological effects beyond oral freshness.

In parallel, several *in vitro* studies have evaluated the cytotoxicity, biocompatibility, wound-healing effects, and antibacterial properties of commercial mouthwash formulations on oral cells (11, 12). However, comparatively little is known about whether these formulations directly modulate inflammatory signaling pathways in resident oral cells independently of their antimicrobial action. In particular, transcriptome-wide analyses addressing cytokine-induced host inflammatory responses are largely lacking. This unresolved question represents an important gap in our understanding of how therapeutic and cosmetic mouth rinses may influence oral inflammatory responses beyond their effects on the biofilm.

Listerine^®^ products represent widely used mouth rinses, with some formulations containing zinc salts (13, 14). Zinc has been recognized as a regulator of inflammatory and immune processes rather than a classical antiseptic (15, 16), with functions as a cofactor for numerous enzymes involved in cellular metabolism, gene regulation, immune function, and tissue repair (17, 18). Zinc homeostasis influences cytokine production, intracellular signaling pathways, oxidative stress responses, and epithelial barrier integrity (16, 19). Transcriptomic analyses have identified metallothionein induction and activation of stress-adaptation pathways as prominent cellular responses to zinc exposure (15, 18–20). Zinc-containing formulations have been evaluated under inflammatory conditions, and experimental studies suggest that zinc salts can influence inflammation-related cellular responses (13). Thus, zinc-containing Listerine^®^ may exert direct host-modulatory effects independently of antimicrobial activity.

Recent single-cell RNA sequencing studies identified fibroblasts as a major source of chemokines in periodontitis (21). Gingival fibroblasts respond to pro-inflammatory cytokines such as interleukin-1β and tumor necrosis factor-α (IL-1β and TNF-α) by producing chemokines, including *CXCL1*, *CXCL2*, *CXCL8*, and *CXCL10*, which promote leukocyte recruitment and amplify inflammatory reactions (22). Oral epithelial cells, such as the HSC2 cell line, also respond to IL-1β and TNF-α by increasing the expression of these chemokines (22). Based on these considerations, we hypothesized that a commercially available Listerine^®^ mouth rinse may exert direct anti-inflammatory effects on gingival fibroblasts and epithelial cells by attenuating cytokine-induced chemokine expression. To test this hypothesis, we established an *in vitro* model using primary human gingival fibroblasts and HSC2 epithelial cells. We evaluated the effects of a zinc-containing Listerine^®^ formulation on inflammatory gene expression and protein release.

## Materials and methods

### Cell culture and cell stimulation

Primary human gingival fibroblasts were isolated from independent healthy donors following written informed consent and approval from the Ethics Committee of the Medical University of Vienna (EK No. 631/2007). The oral squamous cell carcinoma cell line HSC2 was obtained from the Health Science Research Resources Bank (Sennan, Japan). Both cell types were maintained in Dulbecco’s Modified Eagle Medium (DMEM; Sigma-Aldrich, St. Louis, MO, USA) supplemented with 10% fetal bovine serum (FBS; Bio&Sell GmbH, Nuremberg, Germany). The fibroblast medium additionally contained 1% penicillin/streptomycin, whereas the HSC2 medium contained 1% antibiotic-antimycotic solution (both from Sigma-Aldrich). Cells were seeded at 30,000 cells/cm² and incubated at 37 °C in a humidified atmosphere containing 5% CO_2_.

For experiments, cells were cultured in serum-free medium for 3 h and exposed to 5% Listerine^®^ Total Care Gum Protect (Kenvue Inc., Summit, NJ, USA) or to a combination of 10 ng/mL IL-1β and 10 ng/mL TNF-α (both ProSpec-Tany TechnoGene Ltd., Ness-Ziona, Israel) as an inflammatory stimulus. According to the product composition, the undiluted formulation contains zinc chloride (0.09%; approximately 6.6 mM), sodium fluoride (0.022%), and the essential-oil constituents eucalyptol (0.092%), menthol (0.042%), methyl salicylate (0.060%), and thymol (0.064%). Thus, the 5% working dilution corresponded to an estimated zinc chloride concentration of approximately 330 µM. To assess the contribution of individual formulation constituents, cells were incubated with methyl salicylate (2.0%; ReagentPlus^®^, Sigma-Aldrich), thymol (1.8%), eucalyptol (0.3%), or menthol (1.8%) prepared in serum-free medium and applied individually for 3 h under otherwise identical experimental conditions.

### Cell viability assays

Cell viability was assessed following 3 hours of stimulation with serial dilutions (0.6% to 10%) of Listerine^®^ Total Care.

Subsequently, MTT solution (1 mg/mL; 3-[4,5-dimethylthiazol-2-yl]-2,5-diphenyltetrazolium bromide; Sigma-Aldrich, St. Louis, MO, USA) was added to the 96-well plates and incubated for 2 h at 37 °C. The medium was then discarded, and the resulting formazan crystals were dissolved in DMSO (Sigma-Aldrich). Absorbance was measured at 570 nm using a Synergy HTX microplate reader (BioTek Instruments, Winooski, VT, USA), with results normalized to untreated controls. Additionally, cell integrity was confirmed via trypan blue exclusion staining (Sigma-Aldrich).

### RNA isolation, quantitative PCR, and immunoassay

Total RNA was isolated using the GeneMATRIX Universal RNA Purification Kit (EURx^®^, Gdańsk, Poland). Complementary DNA (cDNA) was synthesized from 150 ng total RNA using the LabQ FirstStrand cDNA Synthesis Kit (LabQ, Labconsulting, Vienna, Austria). Quantitative real-time PCR was performed on a CFX Connect™ Real-Time PCR Detection System (Bio-Rad Laboratories, Hercules, CA, USA). Primer sequences are listed in **Supplementary Table 1**. CXCL10 and CXCL8 levels in cell culture supernatants collected after 3 hours of stimulation were measured using DuoSet^®^ ELISA kits (DY266 and DY208, R&D Systems, Minneapolis, MN, USA) at 450 nm by a Synergy HTX microplate reader (BioTek Instruments, Winooski, VT, USA).

### RNA sequencing and data analysis

Three independent libraries were prepared from 500 ng total RNA using the QuantSeq 3′ mRNA-Seq Library Prep Kit FWD (protocol v2) with unique dual indices (Lexogen GmbH, Vienna, Austria). Libraries were amplified using 17 PCR cycles. The quality was assessed using a Bioanalyzer 2100 with a High Sensitivity DNA Kit (Agilent Technologies, Santa Clara, CA), and concentrations were measured using the Qubit dsDNA HS Assay (Invitrogen, Waltham, MA). Pooled libraries were sequenced on a P3 flowcell using a NextSeq 2000 platform (Illumina, San Diego, CA) in 1 × 75 bp single-end mode, generating approximately 10 million reads per sample. FASTQ files were generated using bcl2fastq (v2.19.1.403) and demultiplexed with the Lexogen Idemux tool. Reads were trimmed using cutadapt (v2.8) to remove poly(A) tails, discard reads containing ambiguous bases, and trim bases with Phred quality scores < 30, resulting in approximately 8.9 million reads per sample after processing. Reads were aligned to the human reference genome GRCh38 with GENCODE v29 annotations using STAR (v2.6.1a) (23) in two-pass mode. Gene-level read counts were generated by STAR, and differential expression analysis was performed using DESeq2 (v1.44.0) (24, 25). Sequencing data have been deposited in the GEO repository under accession number GSE319900.

### Bioinformatic analyses

Principal component analysis (PCA) was performed in R (v4.5.2; r-project.org) using normalized gene expression data to evaluate sample clustering, donor variability, and data quality. The prcomp function was used, and the first two principal components were visualized using ggplot2. Heat maps were generated in R (v4.5.2) based on genes with an adjusted *p*-value < 0.05. Volcano plots were also generated by R (v4.5.2) (26), applying a minimum log_2_ fold change of 2 and a −log_10_(*p*-value) threshold of 2.0 (*p* < 0.01). Protein–protein interaction networks were analyzed using the STRING database (27).

### Immunofluorescence

Gingival fibroblasts and HSC2 cells were plated onto Millicell^®^ EZ slides (Merck KGaA, Darmstadt, Germany) at 15,000 cells/cm^2^, followed by overnight starvation in serum-free medium. To assess phospho-STAT1 (pSTAT1) activation and nuclear translocation via immunofluorescence, cells were treated with individual or combined stimuli, including Listerine^®^ Total Care alone, IL-1β/TNF-α alone, or Listerine^®^ in combination with IL-1β/TNF-α or IFN-γ. Recombinant human IFN-γ (100 ng/mL; #300-02, Thermo Fisher Scientific) was used as a positive control. Following a 1-hour stimulation, cells were immediately fixed with 4% paraformaldehyde, permeabilized with 100% methanol (#0082.1, Carl Roth, Karlsruhe, Germany), and blocked with 5% normal goat serum (#5425). The cells were then incubated overnight at 4 °C with a rabbit anti-phospho-STAT1 primary antibody (#9167, 1:400). Signal detection was performed using an Alexa Fluor 488-conjugated goat anti-rabbit secondary antibody (#4412, 1:1000) for 1 hour at room temperature (all from Cell Signaling Technology, Danvers, MA, USA). Images were captured by the Echo Revolve Microscope (Revolve Technologies, San Diego, CA, USA).

### Trace-element quantification

For trace-element quantification, three Listerine^®^ mouth rinse samples (Listerine Total Care Gum Protect, Listerine Total Care Teeth Protect, Listerine Zero) were analyzed by inductively-coupled plasma tandem–mass spectrometry (ICP–MS; Agilent 8800 series instrument attached to an ASX-500 autosampler from Agilent Technologies) under cleanroom conditions. Aliquots of each formulation (0.5 mL) were digested in duplicates with ultrapure nitric acid (20% wt; 3.0 mL) and hydrogen peroxide (30% wt, 0.1 mL) in acid pre-cleaned Teflon^®^ vessels in a microwave oven (Ultrawave Pro from Anton Paar) at 180 °C for 20 min. After cooling to room temperature, digested samples were diluted with ultrapure water (>18.2 MΩ × cm) to a final volume of 10 mL, yielding a matrix compatible with ICP–MS and minimizing carbon/salt load; 14 procedural blanks were prepared identically, replacing Listerine^®^ with ultrapure water (0.5 mL). Quantification was performed by external multi-element calibration covering the expected concentration range of each element, combined with an internal-standard correction (multi-element internal standard solution contained 50 µg/L germanium, rhenium, and yttrium in 3% wt nitric acid; introduced externally at a flow rate of 10% vol relative to the sample flow rate) to compensate for instrumental drift and matrix-related signal suppression/enhancement (**Supplementary Methods S1**).

### Statistical analysis

Data originated from at least 3 independent experiments. Tests were selected based on data distribution and pairing structure. Ratio-paired t-tests were used for paired comparisons, while one-way ANOVA or the Friedman test was applied for multi-group analyses. For targeted qPCR analyses, the absence of multiple-comparison correction is acknowledged as a limitation. Analyses were performed in GraphPad Prism v9 (La Jolla, CA, USA), with significance set at *p* < 0.05. Sex as a biological variable was not analyzed because the study was performed using anonymized primary gingival fibroblasts and established cell lines not designed for sex-based comparisons.

## Results

### Listerine^®^ attenuates cytokine-induced transcriptional reprogramming in gingival fibroblasts

We first established an experimental concentration of Listerine^®^ that allowed assessment of host-cell transcriptional responses while maintaining acceptable cell viability. Based on dose-response experiments, 5% Listerine^®^ was selected because it reduced IL-1β/TNF-α-induced chemokine expression while causing only moderate effects on cell viability (**Supplementary Figures S1** and **S2**). This concentration was therefore used for transcriptomic profiling of primary human gingival fibroblasts exposed to IL-1β/TNF-α in the presence or absence of Listerine^®^.

**Figure 1:**
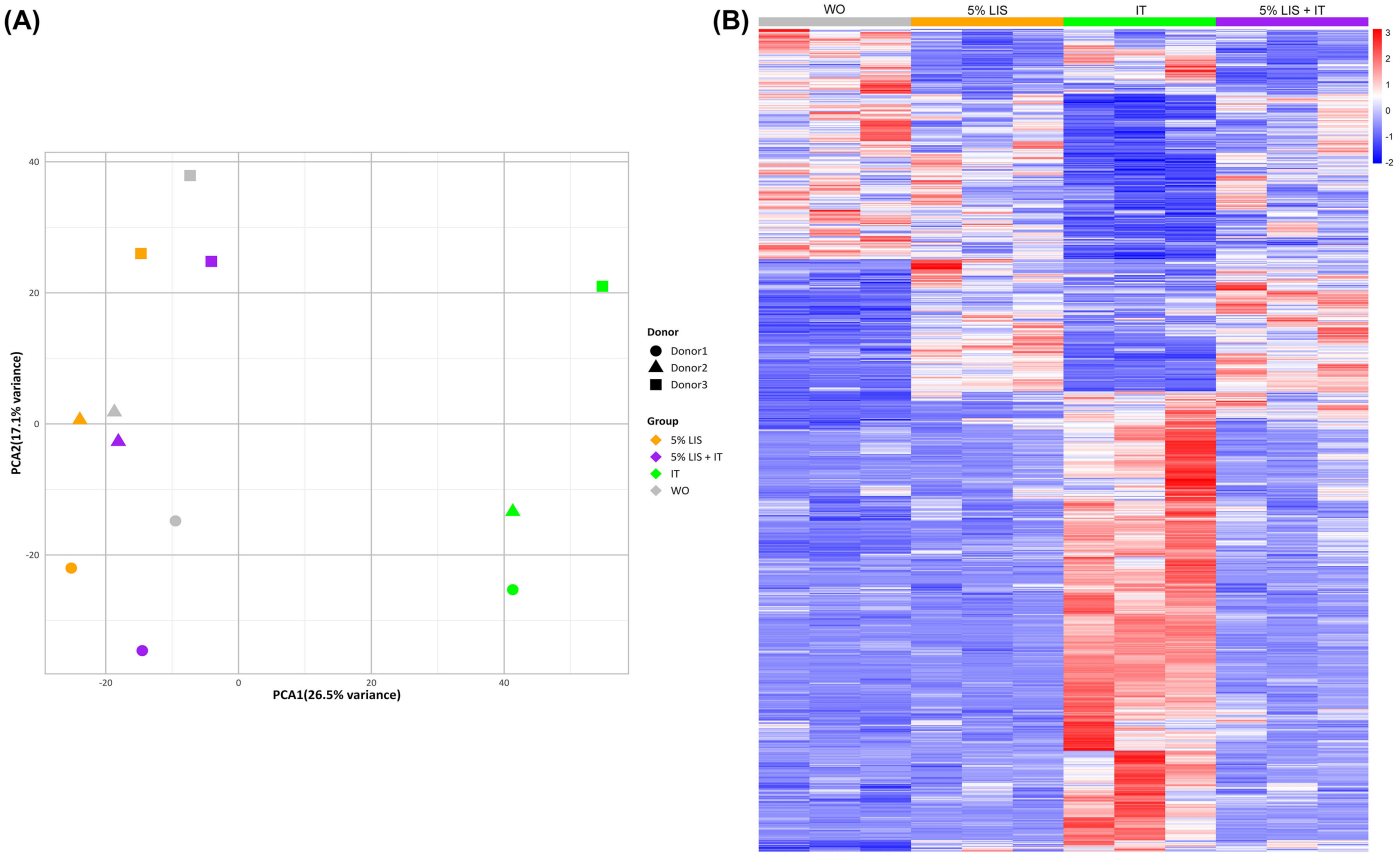
Listerine^®^ attenuates cytokine-induced transcriptional shifts in gingival fibroblasts. **(A)** Principal component analysis (PCA) of RNA-seq data from gingival fibroblasts treated with untreated control (WO), Listerine^®^ (5% LIS), IL-1β/TNF-α (IT), or both (5% LIS+IT). PCA1 (26.5%) and PCA2 (17.1%) demonstrate that the pronounced transcriptional shift triggered by IL-1β/TNF-α is substantially mitigated by LIS co-treatment. Distinct symbols represent individual donors. **(B)** Heat map analysis of differentially expressed genes (|log_2_FC| ≥ 1, *p*adj < 0.05). Rows show relative expression (z-score); columns show treatment groups (WO, untreated control; LIS, Listerine^®^; IT, IL-1β/TNF-α; LIS + IT, co-treatment). Listerine^®^ partially normalized IL-1β/TNF-α-induced inflammatory gene clusters toward baseline levels while preserving donor-dependent variability.

Following bulk RNA sequencing, principal component analysis showed a clear separation of IL-1β/TNF-α-stimulated cells from untreated controls along PC1, indicating a robust cytokine-induced transcriptional shift (**Figure 1A**). Donor-dependent variation was mainly reflected along PC2. Co-treatment with Listerine^®^ shifted the transcriptional profile toward untreated and Listerine^®^-alone conditions, suggesting partial normalization of the cytokine-driven response. This pattern was also evident in the heat map of differentially expressed genes, where Listerine^®^ reduced broad inflammatory gene clusters induced by IL-1β/TNF-α while preserving donor-dependent variability (**Figure 1B**).

**Figure 2:**
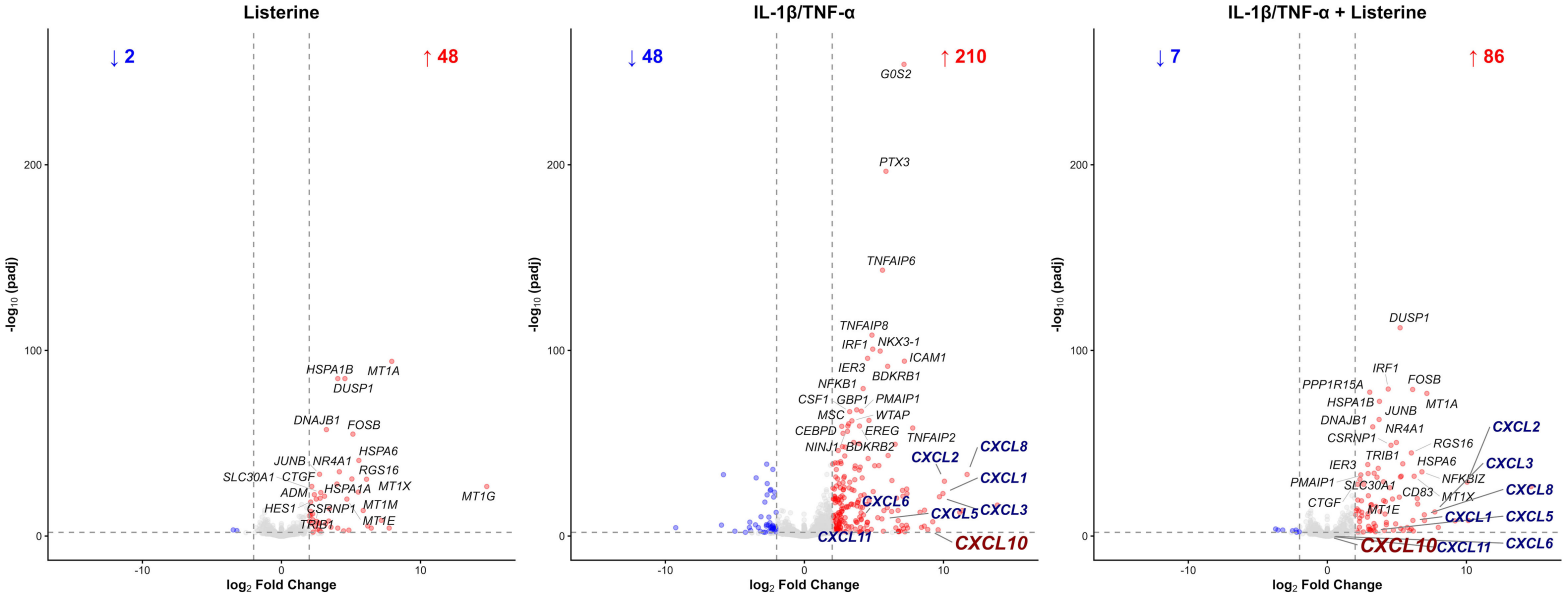
Volcano plot analysis demonstrates broad attenuation of cytokine-induced transcriptional responses by Listerine^®^ in gingival fibroblasts. Volcano plots representing differentially expressed genes (DEGs) in gingival fibroblasts across treatment conditions (|log_2_FC| ≥ 2; *p* < 0.01). Red and blue dots denote significantly up- and down-regulated genes, respectively. Listerine vs. wo: Listerine^®^ monotherapy induces a distinct set of genes, prominently featuring the metallothionein family (e.g., *MT1E*, *MT1X*). IL-1β/TNF-α vs. untreated control (WO): Stimulation with IL-1β/TNF-α induces a robust inflammatory response with numerous DEGs. Listerine + IL-1β/TNF-α vs. WO: Co-treatment with Listerine^®^ substantially reduces the number of significantly regulated genes compared to IL-1β/TNF-α alone, indicating a global dampening of the cytokine-driven transcriptional response.

Volcano plot analysis confirmed that IL-1β/TNF-α induced a broad inflammatory transcriptional program, with 210 significantly upregulated and 48 downregulated genes using the predefined threshold of |log_2_FC| ≥ 2 and *p* < 0.01 (**Figure 2**; **Supplementary Table 2**). The induced genes included cytokines and inflammatory mediators such as *IL6*, *TNF*, *PTGS2*, and *PTGES*, chemokines including *CCL2*, *CCL5*, *CCL7*, *CCL20*, *CXCL1*, *CXCL2*, *CXCL3*, *CXCL5*, *CXCL6*, *CXCL8*, *CXCL10*, and *CX3CL1*, as well as adhesion and immune-modulatory molecules including *ICAM1*, *VCAM1*, *CD274*, *ESM1*, *PTX3*, and *F3*. Negative feedback regulators such as *TNFAIP3*, *NFKBIA*, *NFKBIE*, *TNIP1*, *TNIP3*, and *SOCS3* were also prominently induced.

Co-treatment with Listerine^®^ markedly reduced the magnitude of this transcriptional response, with only 86 upregulated and 7 downregulated genes remaining above the same threshold (**Figure 2**). The attenuation was particularly evident among interferon-associated transcripts, including *CXCL10*, *CXCL11, GBP1*, *GBP2*, *GBP4*, *IFIT2*, *IFIT3*, *OASL*, and *DDX58*. At a lower threshold of |log_2_FC| ≥ 1.0 and adjusted *p* < 0.05, additional interferon-stimulated genes, including *OAS3*, *ISG20*, *MX1*, *MX2*, *IFIT1–5*, *GBP1–5*, *IRF2*, *IFNAR2*, *IFNGR1*, and *IFNGR2*, were also reduced by Listerine^®^. In contrast, classical NF-κB-associated chemokines remained strongly induced despite partial attenuation. For example, *CXCL1*, *CXCL3*, *CXCL5*, and *CXCL8* showed reduced fold changes under co-treatment conditions but remained highly expressed. Similarly, *CCL20* and *CCL5* displayed only partial reductions. These findings indicate that Listerine^®^ does not uniformly suppress all inflammatory mediators but preferentially attenuates an interferon-associated transcriptional program.

### Network analysis identifies suppressed inflammatory modules and a Listerine^®^-induced stress-adaptation signature

**Figure 3:**
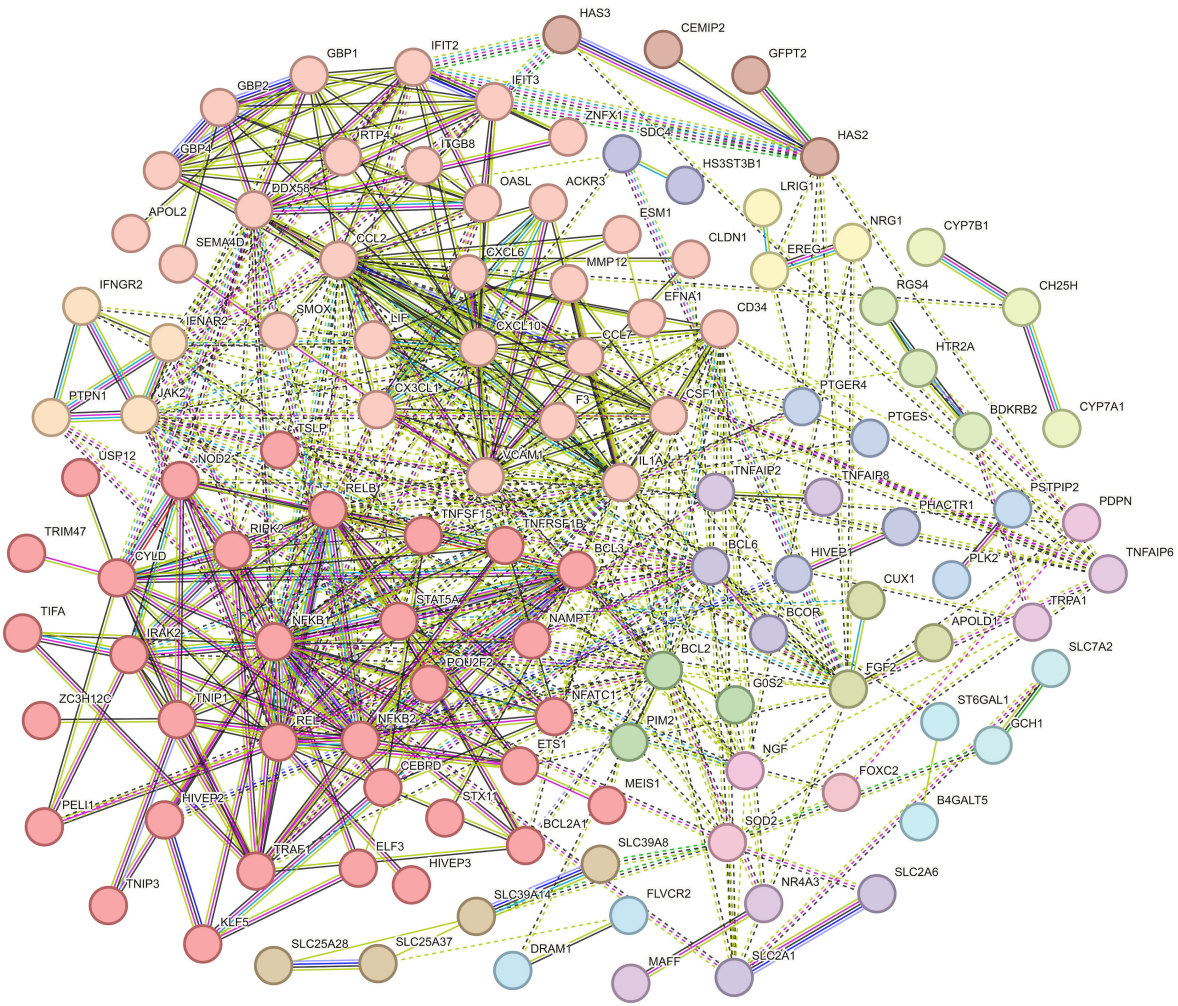
Listerine^®^ suppresses cytokine-induced inflammatory signaling networks in gingival fibroblasts. Protein–protein interaction (PPI) networks were generated using STRING (v11.5) to visualize the dual regulatory effects of Listerine^®^ in gingival fibroblasts. Attenuation of cytokine-induced inflammatory networks. This network comprises genes significantly upregulated by IL-1β/TNF-α but suppressed below significance thresholds by Listerine^®^ co-treatment. Network topology reveals coordinated inhibition of core inflammatory hubs, including chemokines, interferon-induced factors (GBP and IFIT families), and NF-κB pathway components (NFKB1/2, RELB, REL, IRAK2, RIPK2).

To further characterize the transcriptional effects of Listerine^®^, protein–protein interaction networks were generated using STRING analysis. Genes induced by IL-1β/TNF-α but reduced below the significance threshold by Listerine^®^ formed a connected inflammatory network enriched for chemokines, interferon-associated genes, and inflammatory signaling components (**Figure 3**). Central nodes included *CXCL10*, *CXCL6*, *CCL2*, *CCL7*, *GBP* and *IFIT* family members, as well as NF-κB pathway-related molecules such as *NFKB1*, *NFKB2*, *REL*, *RELB*, *IRAK2*, and *RIPK2*. This network structure supports the interpretation that Listerine^®^ attenuates cytokine-amplified inflammatory gene modules, with prominent effects on interferon-associated mediators.

**Figure 4:**
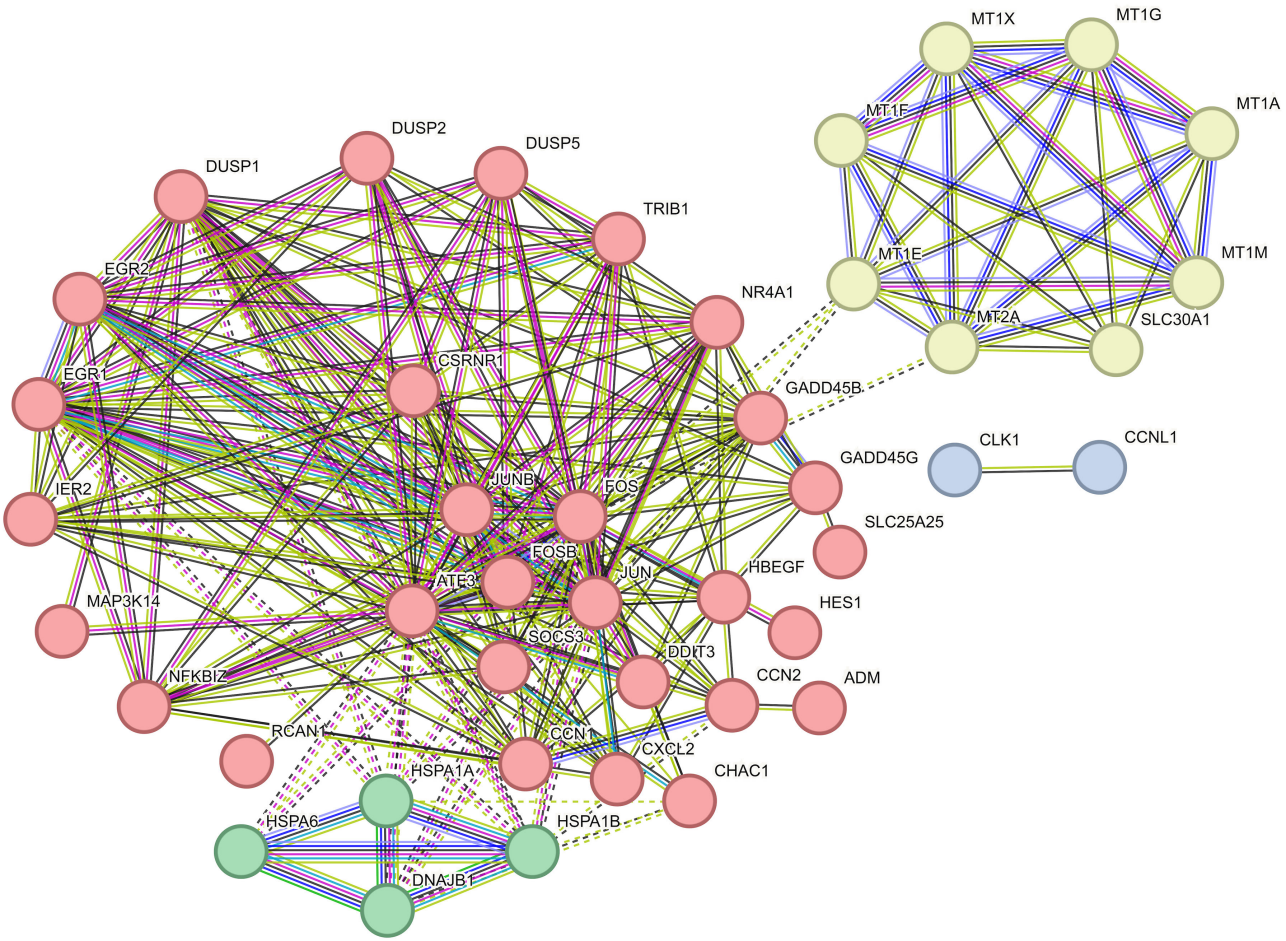
Listerine^®^ alone induces a metallothionein-driven stress-adaptation transcriptional program in gingival fibroblasts. Volcano plot showing differentially expressed genes in gingival fibroblasts treated with Listerine^®^ compared with untreated controls (|log_2_FC| ≥ 2; *p* < 0.01). Upregulated genes were enriched for metallothioneins (*MT1A*, *MT1E*, *MT1F*, *MT1G*, *MT1M*, *MT1X*, *MT2A*) and the zinc transporter *SLC30A1*, consistent with activation of a zinc-buffering and oxidative stress–responsive program. Additional induced genes included immediate-early transcription factors (*FOS*, *FOSB*, *JUN*, *JUNB*, *EGR1*, *EGR2*, *ATF3*, *NR4A1*), signaling regulators (*DUSP1*, *DUSP2*, *DUSP5*, *SOCS3*, *NFKBIZ*, *MAP3K14*), and stress-response genes (*HSPA1A*, *HSPA1B*, *HSPA6*, *DNAJB1*, *DDIT3*, *GADD45B*, *GADD45G*, *CHAC1*), indicating activation of MAPK-, AP-1-, and NF-κB-associated pathways. Only *BMF* and *SALL1* were downregulated (not shown).

In parallel, Listerine^®^ alone induced a distinct transcriptional program dominated by metal- and stress-responsive genes. Volcano plot analysis identified 48 upregulated and 2 downregulated genes following Listerine^®^ treatment alone (**Figure 4**). Upregulated genes included multiple metallothionein family members, such as *MT1A*, *MT1E*, *MT1F*, *MT1G*, *MT1M*, *MT1X*, and *MT2A*, together with the zinc transporter *SLC30A1*. Additional induced genes included immediate-early transcription factors and stress-response regulators, including *FOS*, *FOSB*, *JUN*, *JUNB*, *EGR1*, *EGR2*, *ATF3*, *NR4A1*, *DUSP1*, *DUSP2*, *DUSP5*, *SOCS3*, *NFKBIZ*, *HSPA1A*, *HSPA1B*, *HSPA6*, *DNAJB1*, *DDIT3*, *GADD45B*, *GADD45G*, and *CHAC1*. These findings indicate that Listerine^®^ simultaneously attenuates cytokine-induced inflammatory transcription and induces a zinc-responsive stress-adaptation signature.

### Listerine^®^ preferentially reduces CXCL10 and CXCL11 while inducing metallothioneins in oral cells

**Figure 5:**
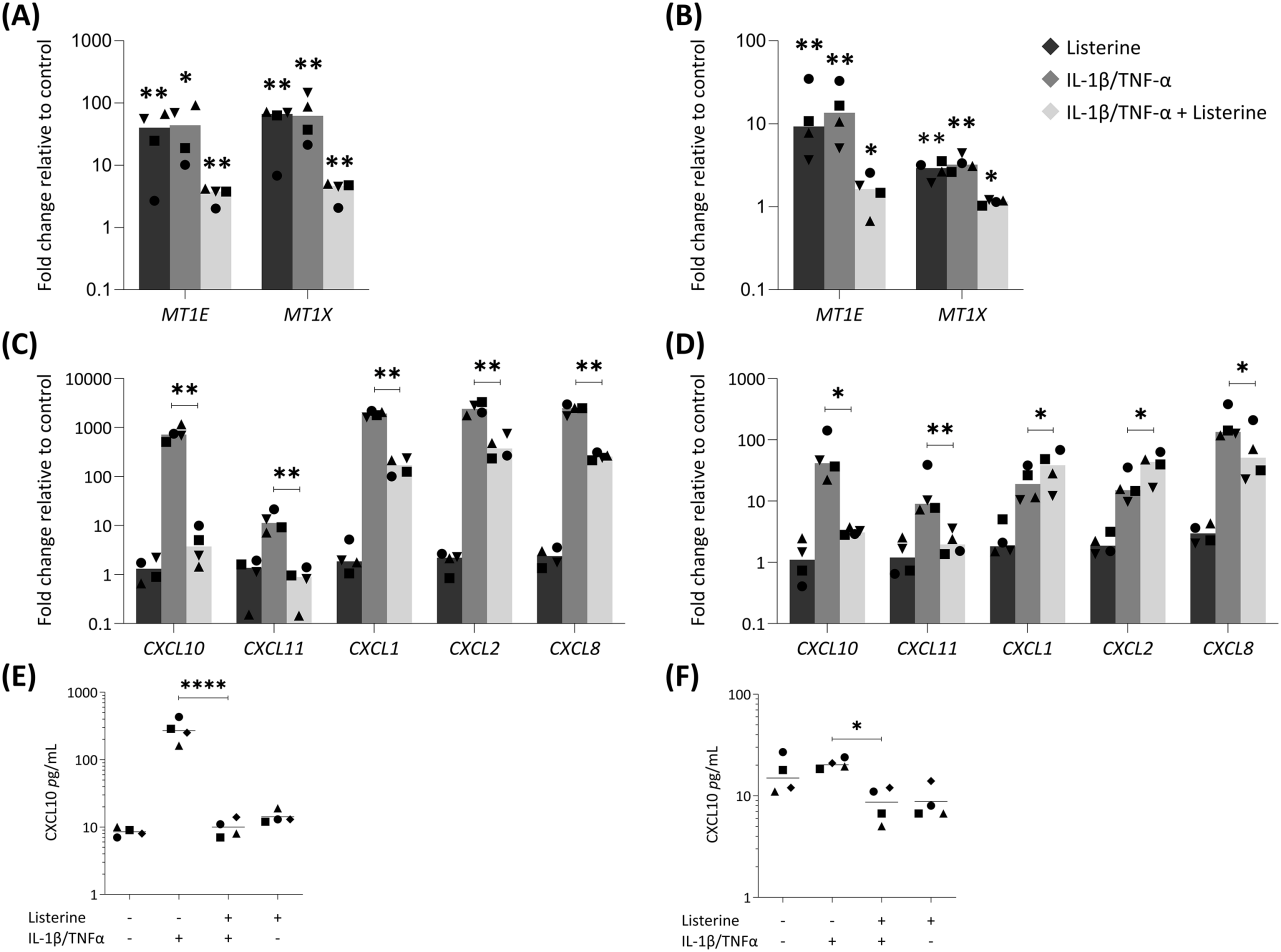
Listerine^®^ upregulates MT expression and exhibits cell-type-specific partial attenuation of chemokines in gingival fibroblasts and HSC2 epithelial cells. **(A, B)** Listerine^®^ induces MT1E and MT1X transcription regardless of IL-1β/TNF-α stimulation in GF **(A)** and HSC2 **(B)** cells, as measured by RT-qPCR. **(C, D)** Relative fold change of CXCL10/11/1/2/8 mRNA expression quantified by RT-qPCR. Listerine^®^ significantly blunts IL-1β/TNF-α-induced chemokine expression in GF cells **(C)**, but only moderately reduces CXCL10/11 levels in HSC2 cells **(D)**. **(E, F)** ELISA quantification of CXCL10 protein levels in culture supernatants after 3 h of stimulation. Listerine^®^ co-treatment significantly reduced cytokine-induced CXCL10 secretion in GF cells **(E)**, whereas the reduction in HSC2 cells was more modest **(F)**. Statistics: 2−ΔΔCt method relative to untreated control (WO=1). Symbols represent 4 independent replicates. P values were calculated using a ratio-paired t-test. *p < 0.05, **p < 0.01.

To validate the transcriptomic findings, selected inflammatory mediators and metallothioneins were analyzed by quantitative PCR in gingival fibroblasts and HSC2 epithelial cells. Listerine^®^ consistently induced *MT1E* and *MT1X* expression in both cell types, either alone or in the presence of IL-1β/TNF-α (**Figures 5A, B**). This confirmed that the formulation activates a conserved metallothionein response across oral stromal and epithelial cells.

Among inflammatory mediators, *CXCL10* and *CXCL11* were particularly sensitive to Listerine^®^. In gingival fibroblasts, Listerine^®^ markedly reduced IL-1β/TNF-α-induced *CXCL10* and *CXCL11* expression, consistent with the RNA-seq data (**Figure 5C**). This pattern was supported by reduced expression of additional interferon-associated genes, including *IFIT2*, *IFIT3*, *GBP1*, and *GBP4* (**Supplementary Figure 3**). In contrast, classical NF-κB-associated chemokines such as *CXCL1*, *CXCL2*, and *CXCL8* were less strongly affected. In HSC2 epithelial cells, Listerine^®^ also induced metallothionein expression and reduced *CXCL10* and *CXCL11*, whereas *CXCL1*, *CXCL2*, and *CXCL8* remained largely unchanged (**Figure 5D**). These data identify *CXCL10* and *CXCL11* as sensitive readouts of the Listerine^®^-modulated inflammatory response.

To determine whether individual essential-oil constituents could reproduce the anti-inflammatory effect of the complete formulation, gingival fibroblasts were stimulated with thymol, eucalyptol, menthol, or methyl salicylate at the indicated concentrations. None of these individual compounds significantly reduced cytokine-induced *CXCL10*, *CXCL1, CXCL2*, or *CXCL8* expression, nor did they significantly alter CXCL8 protein levels (**Supplementary Figure 4**). Methyl salicylate showed a modest trend toward reduced *CXCL10* expression, consistent with the reported anti-inflammatory potential of salicylates (28); however, this effect did not reach statistical significance and is also consistent with observations that neither aspirin nor sodium salicylate suppressed IFN-γ-induced *CXCL10* expression in macrophage-like cells (29). Thus, the observed host-modulatory activity of the complete formulation is unlikely to be attributable to a single isolated essential-oil constituent.

### Zinc is the predominant metal component of the tested formulations

Because metallothionein induction suggested activation of a metal-responsive transcriptional program, trace-element analysis was performed by ICP–MS. Zinc was consistently detected at high concentrations above 400 mg/L across the tested Listerine^®^ formulations, whereas most of the other analyzed elements were below the limit of detection. Besides zinc, only boron, barium, manganese, silver, and thallium were consistently detected, while cadmium, chromium, and vanadium were observed only in selected formulations and at trace levels (**Supplementary Table 3**). These findings identify zinc as the predominant biologically relevant metal component of the formulation. Importantly, zinc alone partially reproduced the suppression of *CXCL10* observed with the complete formulation, although the magnitude of the effect was smaller than that of Listerine^®^, indicating that additional formulation components cannot be excluded (**Supplementary Figure 5**).

### Listerine^®^ does not block canonical STAT1 phosphorylation and nuclear translocation

**Figure 6:**
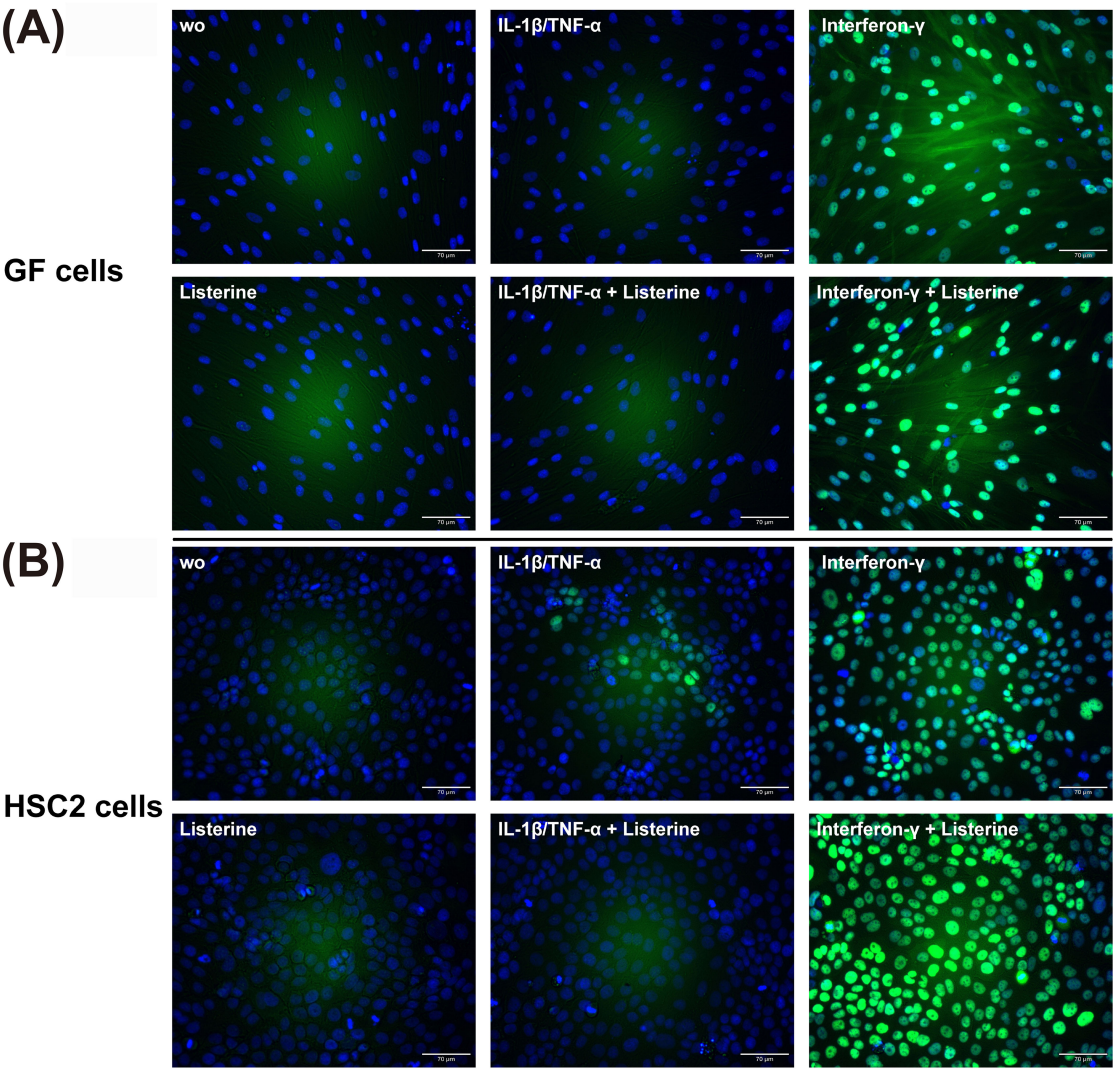
Immunostaining for phospho-STAT1 in gingival fibroblasts and HSC2 epithelial cells. **(A, B)** Interferon-γ (IFN-γ, 100 ng/mL) induced a strong nuclear pSTAT1 signal in both cell types. Treatment with Listerine^®^ did not alter this IFN-γ-induced signal, indicating that Listerine^®^ does not block canonical STAT1 phosphorylation or its nuclear translocation. However, in HSC2 cells, the scattered pSTAT1 signal induced by IL-1β/TNF-α was reduced by Listerine^®^. Scale bar: 70 µm.

Because zinc is not considered a classical direct inhibitor of canonical STAT1 signaling (30), we next examined STAT1 activation by phospho-STAT1 immunostaining. In gingival fibroblasts, IL-1β/TNF-α did not induce an obvious phospho-STAT1 signal, despite inducing *CXCL10*, *CXCL11*, and other interferon-associated transcripts (**Figure 6**). This suggests that these genes are regulated under inflammatory conditions through mechanisms that do not require overt canonical STAT1 activation (31, 32). In HSC2 cells, IL-1β/TNF-α induced a scattered phospho-STAT1 signal that was reduced by Listerine^®^, indicating possible cell type-specific indirect STAT1 activation. Importantly, Listerine^®^ did not visibly reduce IFN-γ-induced nuclear phospho-STAT1 accumulation in either cell type. Thus, Listerine^®^ does not block canonical STAT1 phosphorylation or nuclear translocation, and alternative mechanisms must be considered to explain the reduced expression of interferon-associated inflammatory genes.

## Discussion

In this study, we show that a zinc-containing Listerine^®^ formulation modulates cytokine-induced inflammatory transcription in human oral cells. Rather than acting as a broad suppressor of inflammation, Listerine^®^ preferentially attenuated a subset of interferon-associated inflammatory genes, including *CXCL10*, *CXCL11*, *IFIT* family members, *GBP* family members, and *OASL*. In contrast, classical NF-κB-associated chemokines such as *CXCL1*, *CXCL2*, and *CXCL8* were less strongly affected and remained robustly induced by IL-1β/TNF-α. This pattern was observed in primary gingival fibroblasts and partially conserved in HSC2 epithelial cells, suggesting that oral stromal and epithelial cells can respond to the formulation with a shared but cell-type-specific host-modulatory transcriptional response.

Listerine^®^ preferentially reduced *CXCL10, CXCL11*, and other interferon-associated transcripts without inhibiting IFN-γ-induced STAT1 phosphorylation in gingival fibroblasts or HSC2 cells, indicating that the formulation does not broadly interfere with canonical STAT1 signaling. In contrast, IL-1β/TNF-α induced *CXCL10* and *CXCL11* in the absence of overt STAT1 activation in gingival fibroblasts, while only a scattered phospho-STAT1 signal was observed in HSC2 cells. These findings suggest that the observed transcriptional effects primarily involve noncanonical inflammatory pathways. Indeed, *CXCL10* and related interferon-associated genes can be induced independently of exogenous interferons through cytokine-driven mechanisms involving NF-κB, IRFs, AP-1, chromatin remodeling, and ISRE-dependent promoter activation (31, 32). Consistent with this concept, the *CXCL10* promoter integrates inflammatory and interferon-associated signals through NF-κB, GAS, and ISRE regulatory elements, requiring cooperative activity of NF-κB, STATs, IRFs, and additional transcriptional regulators for maximal induction (32–34). This promoter architecture may explain why *CXCL10* and *CXCL11* were more sensitive to Listerine^®^ than immediate NF-κB target genes such as *CXCL1, CXCL2*, and *CXCL8*. Taken together, preserved IFN-γ-induced STAT1 phosphorylation together with selective suppression of IL-1β/TNF-α-induced interferon-associated genes suggests that Listerine^®^ primarily affects downstream transcriptional regulation rather than proximal interferon-associated activation.

The identity of the formulation component responsible for this effect remains incompletely resolved. However, several independent observations support a relevant contribution of zinc (35). ICP–MS identified zinc as the predominant metal component of the tested formulations, and Listerine^®^ induced a strong zinc-responsive transcriptional signature, including MT1 and MT2 family members together with the zinc transporter SLC30A1. Importantly, zinc-alone experiments partially reproduced the suppression of *CXCL10* in gingival fibroblasts, whereas individual essential-oil constituents did not significantly attenuate cytokine-induced chemokine expression. Moreover, preliminary unpublished observations from ongoing work suggest that zinc alone can attenuate a broader set of interferon-regulated genes. Together, these findings support zinc as a major contributor to the observed host-modulatory activity, while not excluding additional or synergistic contributions from other formulation components.

The possibility that the metallothionein response contributes to the modulation of noncanonical inflammatory pathways should be discussed cautiously. Metallothioneins are well-established regulators of intracellular zinc homeostasis and redox balance and are increasingly recognized as modulators of immune responses (36). However, given the rapid transcriptional changes observed after 3 hours, it appears unlikely that newly synthesized metallothionein proteins are solely responsible for the early suppression of *CXCL10*, *CXCL11*, *IFITs*, and *GBPs*. Rather, metallothionein induction may primarily serve as a sensitive marker of cellular zinc exposure and stress adaptation (37). Whether metallothioneins directly or indirectly contribute to attenuation of interferon-associated transcription, including ISRE-dependent gene regulation, remains to be tested experimentally.

One possible mechanism is that zinc alters the activity of redox-sensitive transcriptional complexes involved in noncanonical interferon-associated gene induction (38). Several transcription factors implicated in *CXCL10* and *CXCL11* regulation, including STAT proteins, IRF family members, NF-κB, and AP-1 components, are sensitive to changes in intracellular redox state (39). Zinc can influence redox signaling, protein conformation, and cysteine-dependent transcription factor activity (40). Thus, zinc exposure may interfere with the transcriptional competence of ISRE-associated regulatory complexes without preventing STAT1 phosphorylation or nuclear translocation (41). This model reconciles the preserved IFN-γ-induced phospho-STAT1 signal with the reduced IL-1β/TNF-α-induced expression of *CXCL10*, *CXCL11*, *IFIT2/3*, *GBP1/4*, and *OASL*.

From an oral immunology perspective, selective attenuation of *CXCL10* and *CXCL11* may be biologically relevant (42). These chemokines contribute to the recruitment of CXCR3-positive immune cells and have been implicated in T-cell-associated inflammatory mucosal diseases, including oral lichen planus and oral mucositis (43). In established periodontitis, *CXCL10* and *CXCL11* produced by gingival fibroblasts are unlikely to represent the dominant drivers of tissue destruction (44). However, modulation of interferon-associated chemokine networks may still influence local immune cell recruitment and inflammatory amplification (45). The clinical relevance of this effect remains uncertain and will require *in vivo* validation, particularly under realistic exposure conditions.

Several limitations should be acknowledged. The experiments were performed in isolated primary gingival fibroblasts and HSC2 epithelial cells, which do not reproduce the cellular complexity of the oral mucosa, including immune cells, endothelial cells, microbiota, extracellular matrix interactions, and mechanical stress. Therefore, the present findings should be interpreted as cell-specific mechanistic observations rather than direct evidence of *in vivo* efficacy. Moreover, although zinc was identified as the predominant metal component, zinc alone partially reproduced CXCL10 suppression, and individual essential-oil constituents were inactive, the relative contribution of zinc and potential synergistic effects with other formulation components remain to be fully resolved. Ongoing zinc-only transcriptomic and mechanistic studies will be important to determine whether zinc alone accounts for the broader suppression of interferon-associated genes observed with the complete formulation.

A further limitation is that the exposure conditions were designed for mechanistic transcriptomic analysis rather than to mimic routine mouth-rinse use. Clinically, Listerine^®^ is applied undiluted for approximately 30 seconds to 2 minutes, whereas we used a 5% dilution for 3 hours to allow measurable transcriptional responses while maintaining cell viability. Thus, the findings demonstrate that the formulation can modulate inflammatory signaling under controlled conditions, but they do not prove equivalent effects during clinical use. Future washout experiments using brief undiluted exposure followed by cytokine stimulation will be important to determine whether transient contact is sufficient to induce sustained suppression of *CXCL10, CXCL11*, and related inflammatory mediators.

The present findings should also be considered in the context of existing clinical evidence for essential oil-containing mouth rinses. Randomized clinical trials and systematic reviews have consistently demonstrated that Listerine^®^ and related essential oil formulations reduce plaque accumulation, gingival inflammation, and bleeding on probing when used as adjuncts to mechanical plaque control (4, 5, 8, 13). These beneficial effects have generally been attributed to antimicrobial activity and the resulting reduction in bacterial biofilm. However, the possibility that these formulations also directly modulate host inflammatory responses has received considerably less attention. Our findings suggest that, in addition to antimicrobial effects, a zinc-containing Listerine^®^ formulation can directly influence cytokine-induced inflammatory transcription in oral stromal and epithelial cells. Although the present *in vitro* observations cannot establish a causal contribution to the clinical benefits reported for mouth rinses, they provide a mechanistic framework that may help explain why these formulations consistently reduce inflammatory parameters in clinical studies. Future clinical investigations should determine whether biomarkers such as CXCL10, CXCL11, or other interferon-associated mediators are similarly modulated *in vivo*.

Collectively, our findings show that a zinc-containing Listerine^®^ formulation attenuates cytokine-induced inflammatory signaling in oral cells, with preferential effects on interferon-associated transcriptional programs. The data support zinc as a major contributor to this host-modulatory activity, while not excluding additional or synergistic contributions from other formulation components. Thus, the present study provides evidence that oral formulations can directly modulate host inflammatory signaling beyond their antimicrobial activity, although the translational relevance of this effect requires validation under clinically realistic exposure conditions.

## Data Availability

The datasets presented in this study can be found in online repositories. The names of the repository/repositories and accession number(s) can be found in the article/**Supplementary Material**.

## References

[1] Kinane DF Stathopoulou PG Papapanou PN . Periodontal diseases. Nat Rev Dis Primers. (2017) 3:17038. doi: 10.1038/nrdp.2017.38 28805207

[2] Elting LS Cooksley C Chambers M Cantor SB Manzullo E Rubenstein EB . The burdens of cancer therapy. Clinical and economic outcomes of chemotherapy-induced mucositis. Cancer. (2003) 98:1531–9. doi: 10.1002/cncr.11671 14508842

[3] Villa A Lodolo M Sonis S . Oral mucosal toxicities in oncology. Expert Opin Pharmacother. (2025) 26:481–9. doi: 10.1080/14656566.2025.2464106 39927612

[4] Tartaglia GM Kumar S Fornari CD Corti E Connelly ST . Mouthwashes in the 21(st) century: a narrative review about active molecules and effectiveness on the periodontal outcomes. Expert Opin Drug Delivery. (2017) 14:973–82. doi: 10.1080/17425247.2017.1260118 27835926

[5] van Swaaij BWM Van der Weijden GA Smith RJ Timmerman MF Slot DE . Essential oils mouthwash with or without alcohol in relation to effect on parameters of plaque and gingivitis: A systematic review and meta-analysis. Int J Dent Hyg. (2025) 23:186–202. doi: 10.1111/idh.12843 39133629 PMC11717972

[6] Van Strydonck DA Slot DE Van der Velden U Van der Weijden F . Effect of a chlorhexidine mouthrinse on plaque, gingival inflammation and staining in gingivitis patients: a systematic review. J Clin Periodontol. (2012) 39:1042–55. doi: 10.1111/j.1600-051x.2012.01883.x 22957711

[7] Jones CG . Chlorhexidine: is it still the gold standard? Periodontol 2000. (1997) 15:55–62. doi: 10.1111/j.1600-0757.1997.tb00105.x 9643233

[8] Fine DH Furgang D Barnett ML Drew C Steinberg L Charles CH . Effect of an essential oil-containing antiseptic mouthrinse on plaque and salivary Streptococcus mutans levels. J Clin Periodontol. (2000) 27:157–61. doi: 10.1034/j.1600-051x.2000.027003157.x 10743861

[9] Vlachojannis C Al-Ahmad A Hellwig E Chrubasik S . Listerine(R) products: an update on the efficacy and safety. Phytother Res. (2016) 30:367–73. doi: 10.1002/ptr.5555 26931615

[10] Yazicioglu O Ucuncu MK Guven K . Ingredients in commercially available mouthwashes. Int Dent J. (2024) 74:223–41. doi: 10.1016/j.identj.2023.08.004 37709645 PMC10988267

[11] Yoshikawa K Sekino J Imamura K Ota K Kita D Saito A . *In vitro* effect of mouthrinse containing essential oils on proliferation and migration of gingival epithelial cells. Phytother Res. (2016) 30:1113–8. doi: 10.1002/ptr.5613 27059802

[12] Geraldino CGP Arbilla G da Silva CM Correa SM Martins EM . Understanding high tropospheric ozone episodes in Bangu, Rio de Janeiro, Brazil. Environ Monit Assess. (2020) 192:156. doi: 10.1007/s10661-020-8119-3 32008106

[13] Fine DH Furgang D Sinatra K Charles C McGuire A Kumar LD . *In vivo* antimicrobial effectiveness of an essential oil-containing mouth rinse 12 h after a single use and 14 days' use. J Clin Periodontol. (2005) 32:335–40. doi: 10.1111/j.1600-051x.2005.00674.x 15811048

[14] LeGeros RZ Rohanizadeh R Lin S Mijares D LeGeros JP Charles CH . Dental calculus composition following use of essential-oil/ZnCl2 mouthrinse. Am J Dent. (2003) 16:155–60. 12967067

[15] Hubner C Haase H . Interactions of zinc- and redox-signaling pathways. Redox Biol. (2021) 41:101916. doi: 10.1016/j.redox.2021.101916 33662875 PMC7937829

[16] Maywald M Wessels I Rink L . Zinc signals and immunity. Int J Mol Sci. (2017) 18:2222. doi: 10.3390/ijms18102222 29064429 PMC5666901

[17] Roohani N Hurrell R Kelishadi R Schulin R . Zinc and its importance for human health: An integrative review. J Res Med Sci. (2013) 18:144–57. PMC3724376 23914218

[18] Wessels I Fischer HJ Rink L . Dietary and physiological effects of zinc on the immune system. Annu Rev Nutr. (2021) 41:133–75. doi: 10.1146/annurev-nutr-122019-120635 34255547

[19] Haase H Rink L . Zinc signals and immune function. Biofactors. (2014) 40:27–40. doi: 10.1016/b978-0-12-802168-2.00021-x 23804522

[20] Vitali F Marini S Balli M Grosemans H Sampaolesi M Lussier YA . Exploring wound-healing genomic machinery with a network-based approach. Pharm (Basel). (2017) 10:55. doi: 10.3390/ph10020055 28635674 PMC5490412

[21] Williams DW Greenwell-Wild T Brenchley L Dutzan N Overmiller A Sawaya AP . Human oral mucosa cell atlas reveals a stromal-neutrophil axis regulating tissue immunity. Cell. (2021) 184:4090–104:e15. doi: 10.1016/j.cell.2021.05.013 34129837 PMC8359928

[22] dos Santos Sanches N Imani A Wang L Pacheco Vitoria OA Reinert H Panahipour L . Tannic acid-loaded gellan gum hydrogels reduce *in vitro* chemokine expression in oral cells. Int J Mol Sci. (2025) 26:5578. doi: 10.3390/ijms26125578 40565042 PMC12193560

[23] Dobin A Davis CA Schlesinger F Jrenkow J Zaleski C Jha S . STAR: ultrafast universal RNA-seq aligner. Bioinformatics. (2013) 29:15–21. doi: 10.1093/bioinformatics/bts635 23104886 PMC3530905

[24] Love MI Huber W Anders S . Moderated estimation of fold change and dispersion for RNA-seq data with DESeq2. Genome Biol. (2014) 15:550. doi: 10.1186/s13059-014-0550-8 25516281 PMC4302049

[25] Zhu A Ibrahim JG Love MI . Heavy-tailed prior distributions for sequence count data: removing the noise and preserving large differences. Bioinformatics. (2019) 35:2084–92. doi: 10.1093/bioinformatics/bty895 30395178 PMC6581436

[26] Wickham H . ggplot2: Elegant Graphics for Data Analysis. Springer-Verlag. (2016). doi: 10.1007/978-3-319-24277-4 33239692

[27] Szklarczyk D Kirsch R Koutrouli M Nastou K Mehryary F Hachilif R . The STRING database in 2023: protein-protein association networks and functional enrichment analyses for any sequenced genome of interest. Nucleic Acids Res. (2023) 51:D638–46. doi: 10.1093/nar/gkac1000 36370105 PMC9825434

[28] Yin MJ Yamamoto Y Gaynor RB . The anti-inflammatory agents aspirin and salicylate inhibit the activity of I(kappa)B kinase-beta. Nature. (1998) 396:77–80. doi: 10.1038/23948 9817203

[29] Sakaeda Y Hiroi M Shimojima T Iguchi M Kanegae H Ohmori Y . Sulindac, a nonsteroidal anti-inflammatory drug, selectively inhibits interferon-gamma-induced expression of the chemokine CXCL9 gene in mouse macrophages. Biochem Biophys Res Commun. (2006) 350:339–44. doi: 10.1016/j.bbrc.2006.09.058 17010317

[30] Read SA O'Connor KS Suppiah V Ahlenstiel CLE Obeid S Cook KM . Zinc is a potent and specific inhibitor of IFN-lambda3 signalling. Nat Commun. (2017) 8:15245. doi: 10.1038/ncomms15245 28513591 PMC5442324

[31] Brownell J Bruckner J Wagoner J Thomas E Loo Y-M Gale M Jr . Direct, Interferon-Independent Activation of the CXCL10 Promoter by NF-κB and Interferon Regulatory Factor 3 during Hepatitis C Virus Infection. J Virol. (2014) 88:4811–21. doi: 10.1128/jvi.02007-13 PMC3911583 24257594

[32] Au-Yeung N Horvath CM . Transcriptional and chromatin regulation in interferon and innate antiviral gene expression. Cytokine Growth Factor Rev. (2018) 44:11–7. doi: 10.1016/j.cytogfr.2018.10.003 30509403 PMC6281172

[33] Makuch E Jasyk I Kula A Lipinski T Siednienko J . IFNbeta-induced CXCL10 chemokine expression is regulated by pellino3 ligase in monocytes and macrophages. Int J Mol Sci. (2022) 23:14915. doi: 10.3390/ijms232314915 36499241 PMC9741470

[34] Ruddy MJ Wong GC Liu XK Yamamoto H Kasayama S Kirkwood KL . Functional cooperation between interleukin-17 and tumor necrosis factor-alpha is mediated by CCAAT/enhancer-binding protein family members. J Biol Chem. (2004) 279:2559–67. doi: 10.1074/jbc.M308809200 14600152

[35] Prasad AS . Zinc is an antioxidant and anti-inflammatory agent: Its role in human health. Front Nutr. (2014) 1. doi: 10.3389/fnut.2014.00014 25988117 PMC4429650

[36] Subramanian Vignesh K Deepe GS Jr . Metallothioneins: Emerging Modulators in Immunity and Infection. Int J Mol Sci. (2017) 18:2197. doi: 10.3390/ijms18102197 PMC5666878 29065550

[37] Krezel A Maret W . The biological inorganic chemistry of zinc ions. Arch Biochem Biophys. (2016) 611:3–19. doi: 10.1016/j.abb.2016.04.010 27117234 PMC5120989

[38] Foster M Samman S . Zinc and regulation of inflammatory cytokines: Implications for cardiometabolic disease. Nutrients. (2012) 4:676–94. doi: 10.3390/nu4070676 22852057 PMC3407988

[39] Marinho HS Real C Cyrne L Soares H Antunes F . Hydrogen peroxide sensing, signaling and regulation of transcription factors. Redox Biol. (2014) 2:535–62. doi: 10.1016/j.redox.2014.02.006 24634836 PMC3953959

[40] Maret W . Zinc in cellular regulation: The nature of an intracellular zinc signal. Int J Mol Sci. (2017) 18:228. doi: 10.3390/ijms18112285 29088067 PMC5713255

[41] Souffriau J Timmermans S Vanderhaeghen T Wallaeys C Van Looveren K Aelbrecht L . Zinc inhibits lethal inflammatory shock by preventing microbe‐induced interferon signature in intestinal epithelium. EMBO Mol Med. (2020) 12:e11917. doi: 10.15252/emmm.201911917 32914580 PMC7539219

[42] Kim JW Ahn AH Jung JY Suh CH Han JH Kim HA . Role of chemokines CXCL9, CXCL10, CXCL11, and CXCR3 in the serum and minor salivary gland tissues of patients with Sjögren's syndrome. Clin Exp Med. (2024) 24:133. doi: 10.1007/s10238-024-01401-4 PMC11189950 38900301

[43] Fang J Wang C Shen C Shan J Wang X Liu L . The Expression of CXCL10/CXCR3 and Effect of the Axis on the Function of T Lymphocyte Involved in Oral Lichen Planus. Inflammation. (2019) 42:799–810. doi: 10.1007/s10753-018-0934-0 30467622

[44] Garlet GP . Destructive and protective roles of cytokines in periodontitis: A re-appraisal from conventional concepts to recent findings. J Dent Res. (2010) 89:1349–63. doi: 10.1177/0022034510376402 20739705

[45] Silva TA Garlet GP Fukada SY Silva JS Cunha FQ . Chemokines in oral inflammatory diseases: apical periodontitis and periodontal disease. J Dent Res. (2025) 86:306–19. doi: 10.1177/154405910708600403 17384024

