# Supplementary material for: Transcriptomic profiling reveals selective attenuation of interferon-associated inflammatory signaling and induction of metallothioneins by a zinc-containing oral rinse
Source: Front Immunol. 2026 Jul 27;17:1896916. doi: 10.3389/fimmu.2026.1896916 (PMC13454332; doi:10.3389/fimmu.2026.1896916)
Supplement: Supplementary file 1 [file Table1.docx]

**Listerine® attenuates cytokine-induced inflammation and activates metallothioneins in oral cells**

Xiaoyu Huang, DDS *†, Anna Podesser *, Murat İnanç Cengiz, DDS *, Ronald A. Glabonjat, PhD ‡, Reinhard Gruber, PhD *§, Layla Panahipour, DDS, PhD *

*Supplementary Figure S1. Listerine® does not impair gingival fibroblast viability at the 5% working concentration*

Primary human gingival fibroblasts were exposed to serial dilutions of Listerine®. Cell viability was assessed by MTT assay. At the concentration used for transcriptomic analyses (5%), Listerine® did not significantly reduce metabolic activity compared with untreated controls. Membrane integrity was confirmed by trypan blue exclusion (not shown). The data represent 4 independent biological replicates.

*Supplementary Figure S2. Listerine dose-dependently attenuates cytokine-induced chemokine expression in gingival fibroblasts*

(A–C) RT-qPCR analysis of *CXCL1*, *CXCL2*, and *CXCL8* mRNA expression in gingival fibroblasts. Cells were stimulated with IL-1β/TNF-α in the presence or absence of indicated Listerine® concentrations. The expression level of the untreated cells was set to 1 for calibration. (D) ELISA quantification of CXCL8 protein levels in culture supernatants after 3 h of stimulation. Listerine® co-treatment significantly reduced cytokine-induced chemokine expression, although levels remained elevated relative to baseline. The data represent 4 independent biological replicates. A ratio-paired t-test determined statistical significance for both mRNA and protein levels.

*Supplementary Figure S3. Listerine® suppresses IL-1β/TNF-α-induced expression of GBP and IFIT family genes in gingival fibroblasts*

RT-qPCR analysis of *IFIT2*, *IFIT3*, *GBP1*, and *GBP4* mRNA levels. Listerine® treatment significantly downregulated the expression of these genes following IL-1β/TNF-α stimulation. Data are expressed as fold changes relative to the untreated control (set to 1 for calibration). Symbols represented 4 independent replicates. A ratio-paired t-test determined significance.

*Supplementary Figure S4. Individual essential oil components of Listerine® fail to attenuate cytokine-induced chemokines in gingival fibroblasts*

(A–C) RT-qPCR analysis of *CXCL10*, *CXCL1*, *CXCL2*, and *CXCL8* mRNA levels. Co-treatment with individual active components of Listerine®—eucalyptol (EUC), menthol, methyl salicylate (MeSA), or thymol (THY)—showed no significant reduction in IL-1β/TNF-α-induced levels (all *p* > 0.05). The expression level of the untreated cells was set to 1 for calibration. (D) ELISA quantification of CXCL8 protein. Secretion levels remained statistically indistinguishable between IL-1β/TNF-α stimulation and individual component co-treatments (*p* > 0.05). Symbols represent 4 independent replicates. A ratio-paired t-test determined significance.

*Supplementary Figure S5. ZnCl_2_ suppresses IL-1β/TNF-α-induced expression of CXCL10 in gingival fibroblasts*

RT-qPCR analysis of *CXCL10*. Zinc treatment suppressed the expression of these genes following IL-1β/TNF-α stimulation for 3 h. Data are expressed as fold changes relative to the untreated control (set to 1 for calibration). Symbols represent 4 independent replicates. A ratio-paired t-test determined significance.

*Supplementary Table S1. Primer Sequences for RT-qPCR Amplification*

| Gene Symbol | Forward Primer | Reverse Primer |
| --- | --- | --- |
| *GAPDH* | AGCCACATCGCTCAGACAC | GCCCAATACGACCAAATCC |
| *CXCL1* | TCCTGCATCCCCCATAGTTA | CTTCAGGAACAGCCACCAGT |
| *CXCL2* | CCCATGGTTAAGAAAATCATCG | CTTCAGGAACAGCCACCAAT |
| *CXCL8* | AACTTCTCCACAACCCTCTG | TTGGCAGCCTTCCTGATTTC |
| *CXCL10* | TGCCATTCTGATTTGCTGCC | TGCAGGTACAGCGTACAGTT |
| *CXCL11* | AGTGTGAAGGGCATGGCTA | TCTTTTGAACATGGGGAAGC |
| *GBP1* | AGGAGTTCCTTCAAAGATGTGGA | GCAACTGGACCCTGTCGTT |
| *GBP4* | AGGCTGCTAAAACACAAGCTG | CCCCAGGTAGAGTGACAATCAT |
| *IFIT2* | AAGCACCTCAAAGGGCAAAAC | TCGGCCCATGTGATAGTAGAC |
| *IFIT3* | TCAGAAGTCTAGTCACTTGGGG | ACACCTTCGCCCTTTCATTTC |

*Supplementary Table S3. Trace-element composition of Listerine® formulations determined by ICP–MS*

Elemental concentrations (µg/L) were measured by ICP–MS under clean-trace conditions. Zinc was detected at high concentrations (>4 × 10⁵ µg/L) across all tested formulations. Most other metals were below the limit of detection (LOD), with only trace-level signals detected for selected elements. Values below LOD are indicated accordingly.

| **Element (µg/L)** | **Listerine Total Care**  **Gum Protect** | | **Listerine Total Care**  **Teeth Protect** | | **Listerine Zero** | |
| --- | --- | --- | --- | --- | --- | --- |
| Ag | 0.4 | 0.5 | 0.5 | 0.4 | 0.4 | 0.4 |
| Al | <14 | <14 | <14 | <14 | <14 | <14 |
| As | <1 | <1 | <1 | <1 | <1 | <1 |
| B | 35 | 38 | 35 | 49 | 54 | 33 |
| Ba | 1.0 | 0.7 | 5.3 | 1.2 | 16.8 | 18.4 |
| Be | <0.08 | <0.08 | <0.08 | <0.08 | <0.08 | <0.08 |
| Ca | <1,000 | <1,000 | <1,000 | <1,000 | <1,000 | <1,000 |
| Cd | 0.44 | 0.41 | <0.06 | <0.06 | 0.16 | 0.13 |
| Co | 0.07 | <0.04 | <0.04 | <0.04 | <0.04 | <0.04 |
| Cr | <0.2 | <0.2 | 1.7 | 1.8 | <0.2 | <0.2 |
| Cu | <300 | <300 | <300 | <300 | <300 | <300 |
| Fe | <12 | <12 | 14 | 14 | <12 | <12 |
| K | 1,600 | <800 | 1,600 | 1,400 | 1,600 | <800 |
| Mg | 70 | <60 | <60 | <60 | 210 | <60 |
| Mn | 2.1 | 2.4 | 1.9 | 1.6 | 2.6 | 3.1 |
| Mo | <0.6 | <0.6 | <0.6 | <0.6 | <0.6 | <0.6 |
| Ni | <8 | <8 | <8 | <8 | <8 | <8 |
| Pb | <0.4 | <0.4 | <0.4 | <0.4 | <0.4 | <0.4 |
| Se | <1 | <1 | <1 | <1 | <1 | <1 |
| Ti | <10 | <10 | <10 | <10 | <10 | <10 |
| Tl | 0.18 | 0.17 | 0.04 | 0.03 | 0.12 | 0.12 |
| V | 0.05 | 0.04 | 0.04 | 0.04 | 0.06 | <0.02 |
| Zn | >400,000 | >400,000 | >400,000 | >400,000 | >400,000 | >400,000 |

*Supplementary Methods S1: Trace-Element Quantification - Calibration*

Quality control included calibration verification standards, continuing calibration checks, blank controls run throughout the sequence, and a series of certified reference materials [CRMs: SRM 1643f and SRM 1577c (n = 4 each) from the National Institute for Standards and Technology, U.S. Department of Commerce; and ERM-CE278k, ERM-668 and ERM-BB186 (n = 3 each) from the European Commission Joint Research Centre] treated in the same way as formulations. Element concentrations of aluminium (Al), barium (Ba), beryllium (Be), boron (B), cadmium (Cd), calcium (Ca), lead (Pb), silver (Ag), thallium (Tl), and zinc (Zn) were measured in no-gas mode; chromium (Cr), cobalt (Co), copper (Cu), iron (Fe), magnesium (Mg), manganese (Mn), molybdenum (Mo), nickel (Ni), and potassium (K) were measured in helium mode (5 mL He/min); and titanium (Ti), vanadium (V), arsenic (As), and selenium (Se) in oxygen reaction mode (30%vol O2) were measured and reported as µg/L in the original samples after applying dilution factors, subtracting digestion blank background, and correction for internal standards and instrument drift. All calibration curves showed a linear correlation of R^2^ ≥ 0.995, and concentrations below the element-specific limit of detection (LOD; calculated as standard deviation × 3.3 of 14 method blanks) were recorded as <LOD. Mean element determination accuracies (min-max) of CRMs were 98% (72-122%) for SRM 1643f, 97% (75-131%) for SRM 1577c, 97% (78-118%) for ERM-CE278k, 99% (77-116%) for ERM-688, and 91% (71-126%) form ERM-BB186; and mean precisions (min-max) were 6.7% (1.2-20%) for SRM 1643f, 7.6% (1.3-23%) for SRM 1577c, 5.2% (0.4-15%) for ERM-CE278k, 5.8% (1.3-12%) for ERM-688, and 5.7% (1.1-17%) for ERM-BB186.
